# Effects of Enzymes or Fermented Feed on Nitrogen Balance, Meat Quality, Intestinal Microbiota Profile and Barrier Functions of Landrace × Rongchang Pigs Fed with a Diversified Low-Protein Diet

**DOI:** 10.3390/vetsci13030219

**Published:** 2026-02-26

**Authors:** Cunji Shui, Jiayao Liao, Jingjing Wang, Zhiru Tang, Renli Qi, Qi Wang, Sishen Wang, Yetong Xu, Zhihong Sun

**Affiliations:** 1 Research Center for Bio-Feed and Molecular Nutrition, College of Animal Science and Technology, Southwest University, Chongqing 400715, China; shuicunji@email.swu.edu.cn (C.S.); 19981750331@163.com (J.L.); w20210205111@email.swu.edu.cn (J.W.); tangzhiru2326@sina.com (Z.T.); 2 Key Laboratory of Chongqing Education Animal Nutrition and Bio-Feed of China, Chongqing 400715, China; 3 National Center of Technology Innovation for Pigs, Chongqing 402460, China; qirenli1982@163.com (R.Q.); wangq0418@126.com (Q.W.); 4 Chongqing Academy of Animal Sciences, Chongqing 402460, China

**Keywords:** biological fermentation, low-protein diet, nitrogen balance, meat quality, Rongchang pigs

## Abstract

Reducing the dependence on traditional protein sources, and decreasing feeding costs and nitrogen emissions, are important tasks in livestock production. A 5 × 5 Latin square nitrogen balance trial (five castrated male pigs) and an animal growth experiment with 120 Landrace × Rongchang pigs were performed and randomly divided into five diets: a normal crude protein level diet (CON); LP diet; diversified LP containing broken rice, rapeseed meal, and DDGS (DLP); DLP + 0.05% cellulase (DLP + CE); and DLP + 20% fermented feed (FDLP). The CON group showed higher nitrogen intake, urinary nitrogen, and total nitrogen excretion than the other four groups (*p* < 0.05). The fecal nitrogen was decreased with the LP, DLP + CE, and FDLP groups compared to the CON group (*p* < 0.05). The mRNA expression of jejunal fatty acid transport protein 1 was upregulated in the LP, DLP + CE, and FDLP groups compared to the CON and DLP groups (*p* < 0.05). The DLP + CE group showed a higher intramuscular fat content in pigs than the CON and DLP groups (*p* < 0.05). In the LD muscle, the FDLP and DLP + CE groups upregulated fatty acid synthase expression compared to the LP and DLP groups (*p* < 0.05). Colonic mRNA expression of zonula occludens-1 and claudin-1 was upregulated in the FDLP group compared to the CON and DLP groups (*p* < 0.05). These results suggest that the supplementation of cellulase and fermented feed in DLP diets improved nitrogen utilization and intestinal health without compromising growth performance or meat quality in Landrace × Rongchang pigs.

## Simple Summary

In livestock farming, reducing the reliance on traditional protein feeds, lowering feeding costs, and cutting down on nitrogen-containing waste are key challenges. This study aimed to investigate if adding enzymes or fermented feed to a low-protein diet with diverse ingredients could help solve these issues while maintaining pig growth and meat quality. We tested five diets on pigs, including a normal-protein diet and diversified low-protein diets with or without added enzymes or fermented feed. The results showed that the diversified low-protein diets with enzymes or fermented feed reduced nitrogen waste and enhanced intestinal health without harming growth or meat quality. This research is valuable because it provides a practical way for farmers to feed pigs more sustainably, lowering costs and environmental impact while keeping livestock healthy.

## 1. Introduction

Low-protein (LP) diets have gained widespread attention in livestock production due to their effects on reducing the dependence on traditional protein sources, decreasing feeding costs and nitrogen emissions from livestock manure, and improving the housing conditions and intestinal health of livestock [1]. For fattening pigs, reducing the crude protein (CP) content within a reasonable range while supplementing balanced amino acids like lysine, tryptophan, threonine, and methionine did not compromise growth performance [2]. Previous studies showed that a 3% reduction in CP content with essential amino acid supply improved the protein digestibility of livestock, and LP diets can further increase tight junction protein expression in growing pigs [3,4]. Moreover, LP diets can improve carcass traits, such as increasing marbling scores and intramuscular fat in the longissimus dorsi (LD) muscle, and improving meat tenderness [5]. However, most of the studies focus on commercial lean-type pig breeds, and the applicability of such LP diet strategies to local crossbred pigs, especially pigs with distinct genetic backgrounds and production characteristics, remains less explored.

Feed costs during the growth and fattening stages are a major expense in pig farming, with the rising prices of corn and soybean meal exacerbating the issue. Reducing feed consumption, improving feed utilization efficiency, and lowering nitrogen emissions are critical goals in the livestock industry. Unconventional feed resources can reduce the reliance on traditional feeds; for instance, corn and soybean meal can be partially replaced by broken rice and distillers’ dried grains with solubles (DDGS) in feed formulations, without showing adverse effects on animal production or intestinal health [6,7]. However, unconventional feeds often have imbalanced nutrition, high crude fiber and anti-nutritional factors, and poor palatability, leading to low utilization rates and potential environmental issues. Techniques like enzyme treatments, microbial fermentation, and feed processing techniques have been used to enhance the utilization efficiency of these feed resources. Enzymes can eliminate anti-nutritional factors, improve digestion, reduce fecal waste, and enhance farming conditions [8]. Some enzymes such as cellulose compensate for endogenous enzyme deficiencies, reduce the crude fiber content of feed, and thus can efficiently improve feed utilization in animals [8,9]. The microbial fermentation of feeds can break down large molecules into absorbable forms, improve protein and lipid contents and reduce crude fiber contents, which are beneficial for the intestinal health of animals [8]. Moreover, fermentation in unconventional feed-based diversified diets can reduce environmental pollution, support sustainable livestock production, advance antibiotic-free farming and address resource limitations, which are highly relevant to the sustainable production of local pig breeds [10].

The Rongchang pig is a renowned local breed in China, predominantly distributed in Sichuan Province and Chongqing city, and it is highly valued for its strong adaptability, tolerance to roughage, disease resistance, meat quality, and genetic stability [11]. The crossbreed of Landrace × Rongchang has proved to be the best for selective breeding because of the high average daily gain (ADG) and feed conversion efficiency of Landrace pigs while retaining the superior meat quality and environmental adaptability of Rongchang pigs [12]. Given the unique characteristics of Landrace × Rongchang pigs and the lack of LP diets for this crossbreed, this study investigates the effects of diversified LP diets, supplemented with cellulose or microbial fermented feed, on nitrogen balance, growth performance, meat quality, and intestinal health in Landrace × Rongchang pigs. We hypothesized that the utilization efficiency of LP diets based on diversified feed ingredients can be improved by using cellulose supplementation and microbial fermentation, while not negatively affecting the growth performance of Landrace × Rongchang pigs. This research will provide evidence for optimizing the LP diet formulations of Landrace × Rongchang pigs and improving sustainable development in the livestock industry.

## 2. Materials and Methods

### 2.1. Animal Use and Care

The project was conducted under the supervision and approval of Institutional Animal Care and Use of Southwest University (animal ethics approval No. IACUC-20250304-37) and the animal procedures followed ethical and animal welfare standards.

### 2.2. Preparation of Feed Fermentation

To prepare the fermentation mixture for the low-protein diet based on diversified feed ingredients (DLP), 40 kg of DLP feed, 24 kg of water, and 20 g of fermentation agent were combined in a plastic fermentation barrel. The mixture was stirred thoroughly, then covered with plastic wrap and a lid. Fermentation lasted for 3 to 4 days until a pleasant alcoholic aroma was detectable. The microbial composition of the fermentation agent is as follows: 3% Bacillus subtilis, 2% Saccharomyces cerevisiae, 2% Lactobacillus, 1.5% Pichia pastoris, and 1% Aspergillus niger (Nanning Weirui Biotechnology Co., Ltd., Nanning, China). The remaining portion consists of defatted rice bran passed through a 30-mesh sieve. The viability of microbial strains in the fermentation agent is as follows: Bacillus subtilis at 3.0 × 10^9^ CFU/g of the fermentation agent; Saccharomyces cerevisiae at 2.0 × 10^9^ CFU/g; Lactobacillus at 2.0 × 10^9^ CFU/g; Pichia pastoris at 1.5 × 10^9^ CFU/g; and Aspergillus niger at 1.0 × 10^9^ CFU/g.

### 2.3. Nitrogen Balance Trial

#### 2.3.1. Experimental Design and Dietary Composition

Five healthy castrated male pigs (Landrace × Rongchang; 25.5 ± 1.00 kg) were selected from Chongqing Qitaijiamu Livestock and Poultry Breeding Co., Ltd. (Chongqing, China) to conduct a nitrogen balance experiment. The study used a 5 × 5 Latin square design, and the five treatment groups were as follows: a diet with normal crude protein (CP) level based on corn–soybean meal (CON), a low-protein diet based on corn–soybean meal (LP), a low-protein diet based on diversified feed ingredients (DLP), a DLP diet + 0.05% cellulase (DLP + CE; cellulase activity ≥ 10,000 U/g), and a DLP diet + fermented feed (FDLP; the ratio of the DLP diet to biological fermented feed in the FDLP group is 4:1 based on dry matter). The formulation of the control group was based on the NRC (2012) guidelines. In the DLP diet, broken rice, rapeseed meal, and DDGS replaced parts of the corn and soybean meal. The LP and DLP diets were supplemented with methionine, threonine, lysine, and tryptophan to ensure that the levels of these four amino acids were consistent with those in the CON diet. The dietary ingredients and composition are presented in Table 1; 0.3% titanium dioxide was added to the feed as an indigestible marker to determine the nutrient digestibility. The pigs were housed individually in stainless steel metabolism cages (1.8 m L × 1.2 m H × 1.0 m W). Throughout the experimental period, each pig was fed three times daily, with feeding times scheduled at 07:30, 13:30, and 19:30 h. Pigs had ad libitum access to fresh water, and daily feed intake was accurately recorded. The room temperature was controlled at 23.0–25.0 °C.

#### 2.3.2. Sample Collection

The experimental period lasted for 7 days, including a 4-day adaptation phase followed by a 3-day sample collection phase. Fecal samples of animals were collected three times daily at 07:00, 13:00, and 17:00 h. Each collection was thoroughly mixed, and approximately 100 g were weighed and subsequently combined with a 10% sulfuric acid solution for fixing nitrogen, maintaining a ratio of feces to dilute sulfuric acid at 10:1. The fecal samples were stored at −20 °C in a freezer. All fecal samples from the same pig were combined and dried at 65 °C for 72 h, and the dried feed samples were ground for further analysis.

The urine was collected for 24 h each day and nylon cloth (0.5 mm pore size) was used for filtration to prevent any feed residue from contaminating the urine. After accurately measuring the total volume of urine collected, an additional 10% sulfuric acid solution was added, corresponding to 10% of the total urine volume, for nitrogen fixation. After that, the urine from the same pig was thoroughly mixed, and a 5% aliquot of the composite sample was stored at −20 °C for further analysis.

#### 2.3.3. Chemical Analysis

The nutrients including crude protein, acid detergent fiber (ADF), neutral detergent fiber (NDF), calcium and phosphorus of the feces, urine, and feed were analyzed following the guidelines of AOAC (2006).

### 2.4. Growth Performance Trial

#### 2.4.1. Experimental Design and Dietary Composition

A total of 120 healthy growing–finishing pigs (Landrace × Rongchang, average weight 31.25 ± 1.22 kg) were selected, consisting of an equal number of males and females. The castrated males and females were randomly assigned to five groups according to a completely randomized block design based on body weight, with six replicates for each group and four pigs in each replicate. The trial lasted 86 days, including a pre-feeding period of 3 days. The experimental diets were formulated for two weight phases: 30–60 kg and 60–100 kg. During the first 47 days (30–60 kg), the five dietary groups were as follows: a corn–soybean meal-based diet (CON, 15.20% CP), a low-protein diet (LP, 13.66% CP), a diversified LP diet (DLP, 13.49% CP), a DLP diet supplemented with 0.05% cellulase (DLP + CE, 13.49% CP), and a DLP diet with bio-fermented feed (FDLP, 13.81% CP). From days 48 to 86 (60–100 kg), the five dietary groups were: a corn–soybean meal-based diet (CON, 12.91% CP), a low-protein diet (LP, 11.38% CP), a diversified LP diet (DLP, 11.14% CP), a DLP diet supplemented with 0.05% cellulase (DLP + CE, 11.15% CP), and a DLP diet with bio-fermented feed (FDLP, 11.48% CP). The dietary ingredients and composition for the two phases are presented in Table 1 and Table 2. The daily feed was provided at 07:30, 13:30, and 19:30 h.

#### 2.4.2. Sample Collection

Throughout the trial, the daily feed consumption was precisely monitored, with pre-fasting body weights at the initiation and termination of each experimental phase. For each stage, the average daily gain (ADG), average daily feed intake (ADFI), and the ratio of feed to gain were calculated. On days 47 and 86, six pigs from each group were selected randomly to collect blood samples via anterior vena cava into EDTA tubes, followed by centrifugation (3000× *g*, 4 °C, and 20 min) for plasma isolation and subsequent storage at −20 °C. On day 86, the pigs were slaughtered by electrical stunning after the blood collection, and the heads, feet, tails, and internal organs were removed to determine carcass weight and calculate the slaughter percentage. An incision was made extending from the first to the tenth rib on the left side to extract the LD muscle, which was utilized to measure meat quality parameters, including shear force, cooking loss, pH value, drip loss, moisture content, inosine monophosphate, intramuscular fat, muscle fatty acid composition, and gene expression. Colonic contents were collected and stored at −80 °C for microbiota and SCFA analysis, while mucosal samples from both the jejunum and colon were frozen in liquid nitrogen then stored at −80 °C for gene expression analysis.

#### 2.4.3. Biochemical Indices

The concentrations of immunoglobulins, hormones, antioxidant capacity indicators, and biochemical indicators in plasma were evaluated using ELISA and biochemical kits. (1) Immunoglobulins: IgG, IgA, and IgM in plasma were assessed utilizing ELISA kits (RX2D711366, RX2D711346 and RX2D711436) sourced from Ruixin Biotechnology Co., Ltd. (Quanzhou, China). (2) Hormones: Growth hormone (GH) and insulin (INS) levels in plasma were measured with ELISA kits (YPJV1667 and SYP-P-174) from Uping Biotechnology Co., Ltd. (Shenzhen, China). (3) Antioxidant capacity indicators including catalase (CAT), glutathione peroxidase (GSH-Px), total antioxidant capacity (T-AOC), total superoxide dismutase (T-SOD), and malondialdehyde (MDA) were measured using kits (A007-1-1, A005-1-2, A015-2-1, A001-3-2 and A003-1-2) from Nanjing Jiancheng Bioengineering Institute (Nanjing, China). (4) Biochemical indicators including blood urea nitrogen (BUN) and aspartate aminotransferase (GOT) were also measured by using kits (C013-2-1 and C010-2-1) from Nanjing Jiancheng Bioengineering Institute (Nanjing, China).

#### 2.4.4. Carcass Traits and Meat Quality

After the pigs were slaughtered, the carcass length and diagonal length were measured using a tape. A vernier caliper was employed to assess the depth at the shoulder, the lumbar–sacral junction, thoracic–lumbar junction, and backfat thickness at the 6th–7th rib interface. The dimensions (height and width) of the LD muscle at the last rib were examined, thereby calculating the loin muscle area. The carcass yield percentage was determined based on the carcass weight and the body weight before slaughter, using the following formula:

Slaughtering percentage (%) = (Carcass weight/Live body weight) × 100%

Loin muscle area (cm^2^) = Height of loin muscle (cm) × Width of loin muscle (cm) × 0.7

After slaughter, the LD muscle on the left side of the carcass was collected and stored at 4 °C for meat quality analysis. The post-mortem pH values at 45 min and 24 h were determined by using a pH meter (HANNA, HI99163, Villafranca Padovana, Italy). The marbling score was assessed using the NPPC standard cards (Bulader Technology Development Co., Ltd., Beijing, China). The meat color parameters (L* for lightness, a* for redness, and b* for yellowness) were determined after blooming for 1 h and the drip loss of the LD muscle was measured by using a sealed plastic bag at 4 °C for 24 h. The cooking loss and shear force of the LD muscle were assessed as described by Kim et al. [9].

#### 2.4.5. Fatty Acid Composition of the LD Muscle

A sample of 0.2 g of the powdered freeze-dried muscle was weighed into an EP tube, and then 1 mL of chloroform–methanol was added for grinding. After that, the samples were centrifuged (14,000 rpm, 3 min) to harvest the supernatant. The supernatant underwent methanolic sulfuric acid treatment (2 mL) with 80 °C water bath incubation (30 min) for esterification. After cooling, 1 mL n-hexane was introduced, followed by 5 min equilibration and final centrifugation (4000 rpm, 7 min). The supernatant was collected and mixed with anhydrous sodium sulfate, then centrifuged for 5 min. After that, the supernatant was diluted threefold, and mixed with methyl salicylate. Finally, an appropriate amount of the sample was put into a detection vial for analysis. The final results will be calculated based on the peak area of the sample and the standard curve.

#### 2.4.6. mRNA Expression of Genes in the Jejunal and Colonic Mucosa and the LD Muscle

Total RNA was extracted from the jejunal and colonic mucosa and LD muscle tissues by using the RNA isolation kit (RC112-01, Vazyme Biotech Co., Ltd., Nanjing, China). Quantification of RNA levels and reverse transcription were performed. The primers for the target genes were designed and confirmed utilizing the NCBI platform (Table S1) and β-actin was selected as the internal control. The mRNA expression levels of the target genes were quantified using the 2^−ΔΔCT^ method.

#### 2.4.7. Colonic Microbiota

Genomic DNA from the colonic contents was extracted and visualized through 1% agarose gel electrophoresis. The barcoded 338F and 806R primers were employed to amplify the bacterial 16S ribosomal RNA in V3–V4 region via PCR. Briefly, the amplification products were analyzed by 2% agarose gel electrophoresis. The PCR products were recovered using the AxyPre DNA Gel Extraction Kit (Axygen Biosciences, Union City, CA, USA) for purification, quantification, and normalization [4]. Sequencing analysis was performed using the Illumina NovaSeq sequencing platform (Shanghai Major Bio-Pharmaceutical Technology Co., Ltd., Shanghai, China). The dada2 plugin was used for sequence quality control, and then sequence clustering and data processing were performed [13].

#### 2.4.8. SCFAs of Colonic Digesta

After weighing 1 g of colonic contents, 1 mL of ultrapure water was added and vortexed. Following centrifugation (15,000 rpm, 15 min), the supernatant was collected and combined with a 25% metaphosphoric acid solution in a 9:1 volume ratio. The MTBE (including internal standard) was added into the solution and vortexed. The solution was filtered with 0.22 µm membrane before analysis. SCFAs were quantified by gas chromatography (Agilent 7890B; Agilent Technologies, Wilmington, DE, USA) using a VF-5ms column (30 m × 0.25 mm × 0.5 μm, 1.0 mL/min column flow) as previously described [13].

### 2.5. Statistical Analysis

All of the data were checked for normality by using the UNIVARIATE procedure of SAS (version 9.2, SAS Inst. Inc., Cary, NC, USA). For the nitrogen balance experiment, data from a 4 × 4 Latin square design were processed via the SAS 9.2 mixed model, which included the fixed effects of dietary treatments and the random effects of animals and time. The individual pig was the experimental unit and multiple comparison analysis was performed by using Tukey’s post hoc test. For the animal growth experiment, the pen was the experimental unit and one-way ANOVA was performed to examine the differences among treatments. Differences in α-diversity indices among groups were analyzed using the Kruskal–Wallis test. The results are expressed as means ± SEM, where *p* < 0.05 indicates a significant difference.

## 3. Results

### 3.1. Nitrogen Balance

Table 3 shows that the five dietary treatments had no effects on the average daily feed intake (ADFI), retained nitrogen (RN), or the ratio of nitrogen retention to nitrogen intake (RN/IN) in Landrace × Rongchang growing pigs (*p* > 0.05). Pigs fed the CON diet had higher nitrogen intake (IN), urinary nitrogen (UN), and total nitrogen excretion (TNE) compared to the other four treatment groups (*p* < 0.05). In comparison to the CON group, the fecal nitrogen content (FN) in the LP, DLP + CE, and FDLP groups of Landrace × Rongchang pigs was reduced (*p* < 0.05).

### 3.2. Growth Performance

As shown in Table 4, during the experimental period from days 1 to 47 (30–60 kg), the final body weight, ADG, ADFI and F/G were not significantly changed by the dietary groups (*p* = 0.06). From days 47 to 86 (60–100 kg), no significant differences were observed in the final body weight, ADG, ADFI and F/G between the dietary groups (*p* > 0.05).

### 3.3. Plasma Biochemical Indices

As shown in Table 5, during the experimental period from days 1 to 47 (30–60 kg), the plasma BUN levels in the LP, DLP, DLP + CE, and FDLP groups were lower than the CON group (*p* < 0.05). The plasma GH levels in the LP group were decreased compared to the CON group (*p* < 0.05), while no differences were noted among the LP, DLP, DLP + CE and FDLP groups (*p* > 0.05). Pigs in the LP group exhibited a higher plasma T-AOC than the CON, DLP, DLP + CE, and FDLP groups (*p* < 0.05). Conversely, pigs in the DLP group showed a reduction in plasma T-AOC compared to the CON group (*p* < 0.05), while no significant differences were observed between the DLP + CE and FDLP groups (*p* > 0.05). The plasma SOD levels in the DLP + CE group were higher compared to the CON, LP, DLP, and FDLP groups (*p* < 0.05), whereas no significant differences were found between the LP, DLP, and FDLP groups (*p* > 0.05). Additionally, plasma MDA levels were reduced in the LP, DLP + CE, and FDLP groups compared with the DLP group (*p* < 0.05). The plasma GSH-Px levels in the FDLP group were higher than the CON, LP, and DLP groups (*p* < 0.01), with no significant difference observed between the DLP + CE and FDLP groups (*p* > 0.05). Moreover, the plasma levels of AST, INS, CAT, IgM, IgA, and IgG were not significantly changed among the dietary treatments (*p* > 0.05).

During the experimental period from days 48 to 86 (60–100 kg), the plasma GH levels in the LP and DLP groups were lower compared to the FDLP group (*p* < 0.05), whereas no significant differences were found in the plasma GH levels of the DLP + CE and FDLP groups compared to the CON group (*p* > 0.05). Furthermore, the plasma levels of BUN, AST, INS, CAT, T-AOC, SOD, MAD, GSH-Px, IgM, IgA, and IgG in Landrace × Rongchang finishing pigs were not significantly changed among the dietary treatments (*p* > 0.05).

### 3.4. Carcass Traits and Meat Quality

Table 6 shows that there were no significant changes in carcass weight, carcass yield, carcass straight length, carcass skew length, loin eye area, back fat thickness at the thickest point of shoulder, back fat thickness at the thoracolumbar junction, back fat thickness at the lumbar–sacral junction, and back fat thickness among the dietary treatment groups (*p* > 0.05).

The pH of the LD muscle at 45 min, colorimetric card rating, meat color L*, meat color a*, meat color b*, drip loss, cooking loss, shear force, and inosine acid content were not significantly changed by the dietary treatments (*p* > 0.05). The pH of the LD muscle at 24 h in the FDLP group was higher than the CON, DLP, and DLP + CE groups (*p* < 0.05), while the pH value did not show significant differences between the LP and FDLP groups (*p* > 0.05). Furthermore, there were no significant changes in the pH at 24 h among the CON, LP, DLP, and DLP + CE groups (*p* > 0.05). The marbling score of the LD muscle in the DLP + CE group was higher than that in the CON and DLP groups (*p* < 0.05), and the marble score in the FDLP group was greater than that in the DLP group (*p* < 0.05). The intramuscular fat content in the LD muscle of the DLP + CE group was higher than the CON and DLP groups (*p* < 0.05), while no differences were found between the LP and FDLP groups when compared to the DLP + CE group (*p* > 0.05).

### 3.5. Long-Chain Fatty Acid Contents in the LD Muscle

As shown in Table 7, there were no differences in the contents of methyl tetradecanoate (C14:0), methyl hexadecanoate (C16:0), methyl cis-9-hexadecenoate (C16:1), methyl heptadecanoate (C17:0), methyl octadecanoate (C18:0), methyl trans-9-octadecenoate (C18:1n9t), methyl cis-9-octadecenoate (C18:1n9c), methyl cis-9,12-octadecadienoate (C18:2n6c), methyl eicosanoate (C20:0), methyl cis-11-eicosenoate (C20:1), methyl cis-9, 12, 15-octadecatrienoate (C18:3n3), methyl cis-11, 14-eicosadienoate (C20:2), methyl cis-8, 11, 14-eicosatrienoate (C20:3n6), methyl cis-5, 8, 11, 14-eicosatetraenoate (C20:4n6), and methyl tricosanoate (C23:0) in the LD muscle among the groups (*p* > 0.05). The content of decanoic acid (C10:0) in the LD muscle was decreased in the DLP group than the LP and DLP + CE groups (*p* < 0.05); however, the C10:0 content was not changed by the LP, DLP, DLP + CE, and FDLP groups compared to the CON group (*p* > 0.05). The content of lauric acid (C12:0) in the LD muscle was increased with the LP group compared with the DLP group (*p* < 0.05), while C12:0 content was not significantly changed among the LP, DLP, DLP + CE, and FDLP groups compared to the CON group (*p* > 0.05).

### 3.6. Gene Expression of Nutrient Transport Carrier in the Jejunal Mucosa

**Figure 1:**
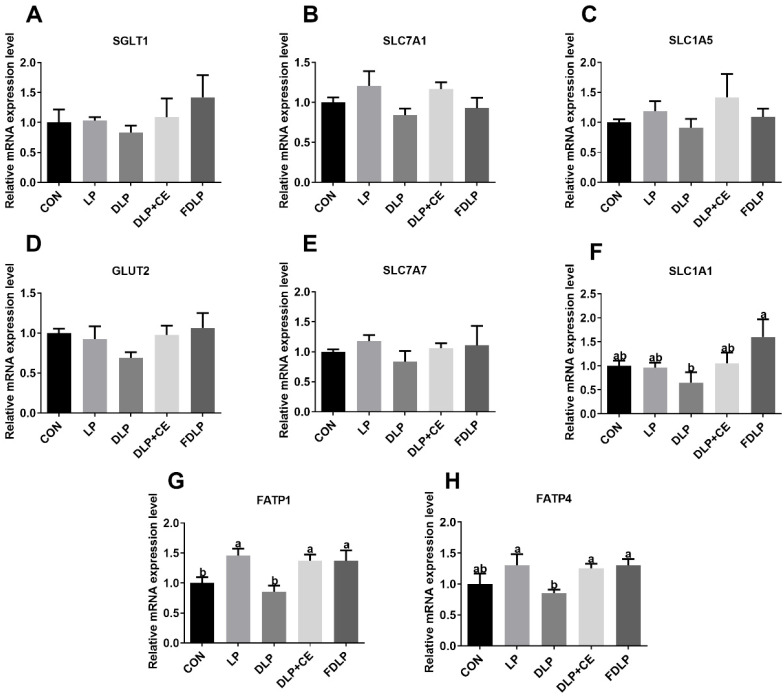
Effects of diversified low-protein diet supplemented with enzyme preparation or fermented feed on the relative expression of nutrient transporter mRNA in jejunum mucosa of Landrace × Rongchang finishing pigs (*n* = 6). (**A**) SGLT1, sodium–glucose linked transporter 1; (**B**) SLC7A1, solute carrier family 7 member 1; (**C**) SLC1A5, solute carrier family 1 member 5; (**D**) GLUT2, glucose transporter type 2; (**E**) SLC7A7, solute carrier family 7 member 7; (**F**) SLC1A1, solute carrier family 1 member 1; (**G**) FATP1, fatty acid transport protein 1; (**H**) FATP4, fatty acid transport protein 4. Data was displayed as means ± SEM in figures. ^a,b^ Different lowercase letters indicated that the results of post hoc analysis were significant (*p* < 0.05). CON, control diet; LP, low-protein diet; DLP, diversified low-protein diet; DLP + CE, diversified low-protein diet with cellulase; FDLP, diversified low-protein diet with fermented feed.

Figure 1 shows that the relative mRNA expression levels of sodium–glucose co-transporter 1 (SGLT1) and glucose transporter 2 (GLUT2) in the jejunal mucosa of pigs were not significantly changed among dietary groups (*p* > 0.05). Similarly, the relative mRNA expression levels of cationic amino acid transporter 1 (SLC7A1), alanine-serine-cysteine transporter 2 (SLC1A5), and solute carrier family 7 member 7 (SLC7A7) also showed no differences among the groups (*p* > 0.05). The relative mRNA expression level of solute carrier family 1 member 1 (SLC1A1) in the jejunal mucosa of the FDLP group was increased compared to the DLP group (*p* < 0.05). The relative mRNA expression levels of fatty acid transport protein 1 (FATP1) in the jejunal mucosa of the fattening pigs from the LP, DLP + CE, and FDLP groups were higher than those in the CON and DLP groups (*p* < 0.05). Additionally, compared to the DLP group, the relative mRNA expression levels of FATP4 in the jejunal mucosa of pigs from the LP, DLP + CE, and FDLP groups were upregulated (*p* < 0.05).

### 3.7. Gene Expression Related to Lipid Metabolism, Protein Synthesis and Muscle Fiber in the LD Muscle

**Figure 2:**
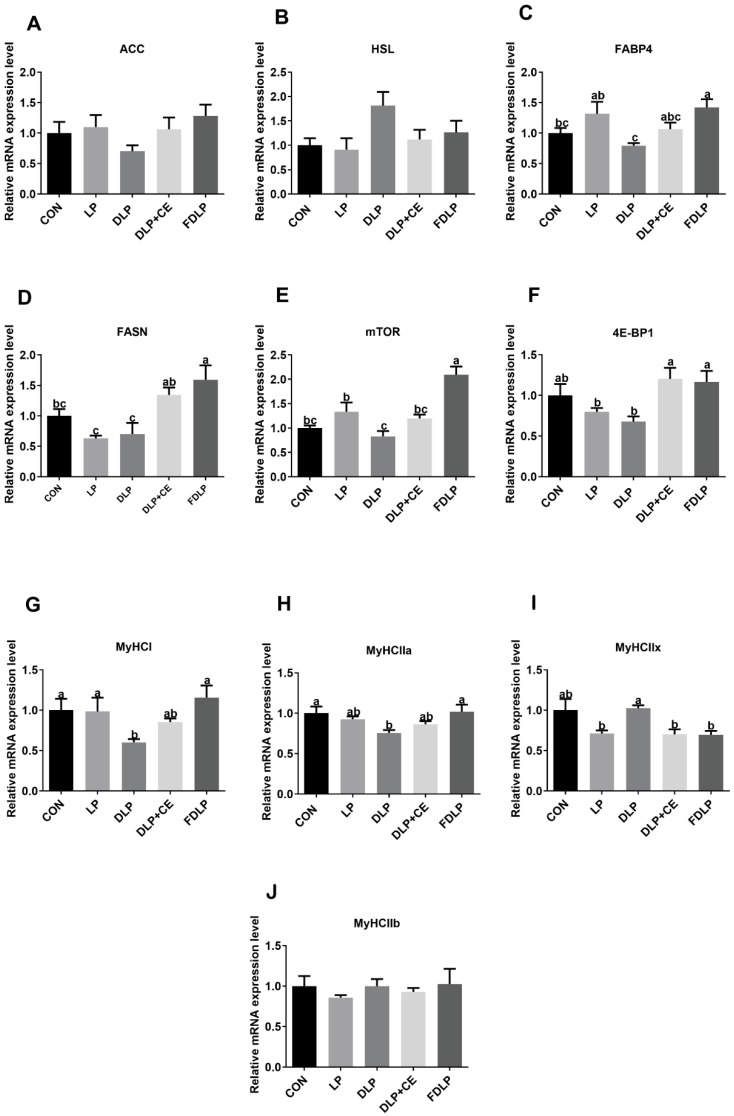
Effects of diversified low-protein diet supplemented with enzyme preparation or fermented feed on the relative mRNA expression of lipid metabolism and protein synthesis-related genes and muscle fiber genes in the longissimus dorsi muscle of Landrace × Rongchang finishing pigs (*n* = 6). (**A**) ACC, acetyl-CoA carboxylase; (**B**) HSL, hormone-sensitive triglyceride lipase; (**C**) FABP4, fatty acid binding protein 4; (**D**) FASN, fatty acid synthase; (**E**) mTOR, mammalian target of rapamycin; (**F**) 4E-BP1, eukaryotic translation initiation factor 4E (eIF4E)-binding protein 1; (**G**) MyHC I, myosin heavy chain 1; (**H**) MyHC IIa, myosin heavy chain IIa; (**I**) MyHC IIx, myosin heavy chain IIx; (**J**) MyHC IIb, myosin heavy chain IIb. Data was displayed as means ± SEM in figures. ^a–c^ Different lowercase letters indicate that the results of post hoc analysis were significant (*p* < 0.05). CON, control diet; LP, low-protein diet; DLP, diversified low-protein diet; DLP + CE, diversified low-protein diet with cellulase; FDLP, diversified low-protein diet with fermented feed.

In Figure 2, there were no differences in the mRNA expression levels of acetyl-CoA carboxylase (ACC) and hormone-sensitive triglyceride lipase (HSL) genes among the LD muscles of the five groups of fattening pigs (*p* > 0.05). Compared to the CON and DLP groups, the mRNA expression level of the FABP4 gene in the LD muscle of the FDLP group was elevated; additionally, the mRNA expression levels of the FABP4 in the LP and FDLP groups were upregulated compared to that in the DLP group (*p* < 0.05). The gene expression level of FASN in the LD muscle of the FDLP group was upregulated compared to the CON, LP and DLP groups; furthermore, the DLP + CE groups exhibited higher relative mRNA expression levels of FASN than the LP and DLP groups (*p* < 0.05). The FDLP group upregulated the mRNA expression level of mTOR in the LD muscle compared to the CON, LP, DLP, and DLP + CE groups (*p* > 0.05), while the mRNA expression level of mTOR in the LP group was elevated compared to the DLP group (*p* < 0.05). The mRNA expression levels of 4E-BP1 in the LD muscle were upregulated in both the FDLP and DLP + CE groups compared to the LP and DLP groups (*p* < 0.05).

Moreover, the gene expression level of MyHC I in the LD muscle of the DLP group was downregulated compared to the CON, LP, and FDLP groups (*p* < 0.05), while the mRNA expression levels of MyHC IIa in the LD muscle were not significantly changed by the LP, DLP + CE, and FDLP groups compared to the CON group (*p* > 0.05); however, the DLP group exhibited a lower gene expression level of MyHC IIa compared to the CON and FDLP groups (*p* < 0.05). The gene expression levels of MyHC IIx in the LD muscle of the LP, DLP + CE, and FDLP groups were downregulated compared to the CON group (*p* < 0.05). However, no significant changes were observed in the mRNA expression levels of the MyHC IIb gene among the five groups of fattening pigs (*p* > 0.05).

### 3.8. Microbiota Community and SCFA Composition in the Colon

The number of shared Amplicon Sequence Variants (ASVs) in the colonic digesta of fattening pigs across the five experimental groups was 166 (Figure S1A). The CON group exhibited 11 unique ASVs, while the LP group had 9 unique ASVs. The DLP group contained 7 unique ASVs, and the DLP + CE group had 5 unique ASVs. Notably, the FDLP group displayed 32 unique ASVs. As the amount of randomly sampled sequencing data increased, the diversity index (Shannon) curves for the colonic digesta of the fattening pigs gradually leveled off, indicating that the sequencing data volume for this dilution curve experiment was sufficient and the sequencing results are reliable for subsequent analyses (Figure S1B). There were no significant changes in the ACE index, Shannon index, Chao1 index, and Coverage index among the microbial communities in the colonic digesta of the fattening pigs across the various groups (Figure S1C–F, *p* > 0.05).

**Figure 3:**
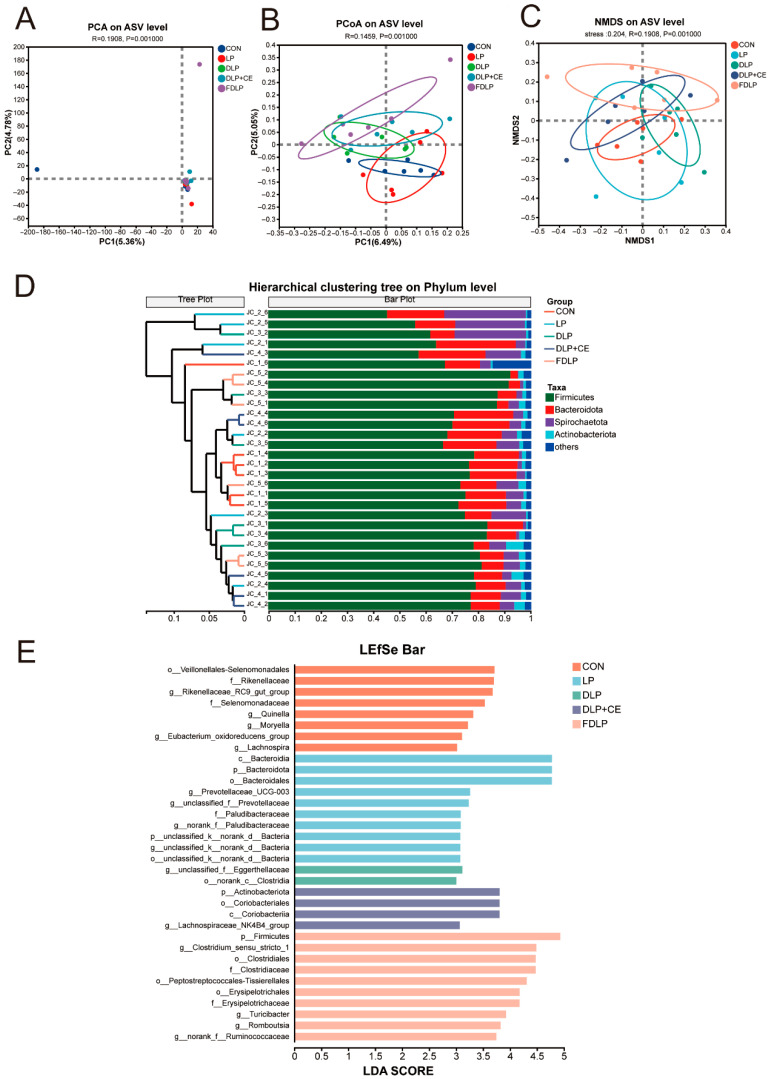
Effects of diversified low-protein diet supplemented with enzyme preparation or fermented feed on colon microbial profile of Landrace × Rongchang finishing pigs (*n* = 6). (**A**) The principal component analysis (PCA) plot. (**B**) The principal coordinate analysis (PCoA) plot. (**C**) The non-metric multidimensional scaling (NMDS) plot. (**D**) The Unweighted Pair-group Method with Arithmetic Means (UPGMA) plot. (**E**) The LEfSe analysis (LDA score > 3). CON, control diet; LP, low-protein diet; DLP, diversified low-protein diet; DLP + CE, diversified low-protein diet with cellulase; FDLP, diversified low-protein diet with fermented feed.

As shown in Figure 3A–C, the microbial communities from the FDLP group had distinct clusters compared to the CON and LP groups. The Unweighted Pair-group Method with Arithmetic Means (UPGMA) presents a hierarchical clustering tree that reflects the similarities and differences in species composition among samples. The microbial communities within the FDLP and DLP + CE groups exhibited higher intra-group similarity, characterized by shorter branch lengths (Figure 3D). As shown in Figure 3E, the CON group was enriched with Rikenellaceae, Rikenellaceae_RC9_gut_group, and Selenomonadaceae. The LP group was enriched with Bacteroidia, Bacteroidota, and Prevotellaceae_UCG-003. In the DLP group, the microbiota including unclassified_f_Eggerthella and norank_c_Clostridia were enriched. The enriched microbiota in the DLP + CE group were Actinobacteriota, Coriobacteriia and Lachnospiraceae_NK4B4_group. The enriched microbiota in the FDLP group were Firmicutes, Erysipelotrichales, Clostridium sensu stricto 1, Turicibacter and Romboutsia.

At the phylum level, the dominant microbiota in the colonic digesta of fattening pigs across the five groups was the Firmicutes and Bacteroidetes, followed by Spirochaetota and Actinobacteriota (Figure S2A and Table S2). The relative abundance of Firmicutes was increased with the FDLP and DLP + CE groups compared with the LP group (*p* < 0.05). The relative abundance of Bacteroidetes was increased with the LP group compared with the CON group (*p* < 0.05). Compared to the DLP group, the relative abundance of the Peptostreptococcaceae was reduced with the CON and LP groups (Figure S2B and Table S3, *p* < 0.05). The relative abundance of Clostridiaceae was increased with the DLP group compared with the CON, LP, DLP + CE, and FDLP groups (*p* < 0.05). At the genus level, the relative abundance of Clostridium sensu stricto 1 in the DLP group was higher compared with the CON, LP, DLP + CE, and FDLP groups (Figure S2C and Table S4, *p* < 0.05).

The acetate concentration in the colonic content of fattening pigs in the FDLP group was higher than the DLP group (*p* < 0.05), while the acetate concentration in the colonic content of fattening pigs in the DLP group was lower than the CON group (Table 8, *p* < 0.05). The propionate concentration in the colonic content of the DLP group was lower than the CON, LP, and FDLP groups (*p* < 0.05). Compared to the LP and DLP group, the valerate concentration in the colonic content of fattening pigs in the DLP + CE and FDLP groups was increased (*p* < 0.05). There were no differences in the concentrations of isobutyrate, butyrate, and isovalerate in the colonic content of fattening pigs among the different groups (*p* > 0.05).

### 3.9. Gene Expression of Tight Junction Proteins in the Colonic Mucosa

**Figure 4:**
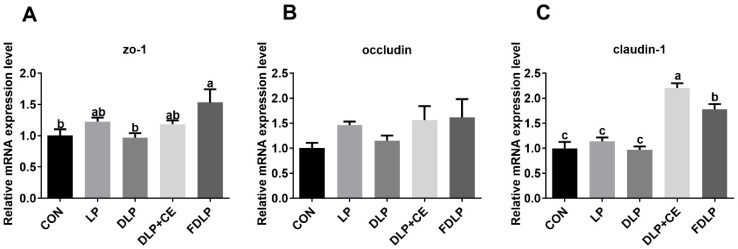
Effects of diversified low-protein diet supplemented with enzyme preparation or fermented feed on mRNA relative expression levels of colon mucosal barrier function-related genes in Landrace × Rongchang finishing pigs (*n* = 6). (**A**) ZO-1, zonula occludens protein-1; (**B**) occluding protein; (**C**) claudin-1 protein. Data was displayed as means ± SEM in figures. ^a–c^ Different lowercase letters indicate that the results of post hoc analysis were significant (*p* < 0.05). Abbreviations: CON, control diet; LP, low-protein diet; DLP, diversified low-protein diet; DLP + CE, diversified low-protein diet with cellulase; FDLP, diversified low-protein diet with fermented feed.

As shown in Figure 4, the colonic mRNA expression of zonula occludens protein-1 (ZO-1) of fattening pigs in the FDLP group was upregulated compared with the CON and DLP groups (*p* < 0.05). The gene expression of claudin-1 in the DLP + CE and FDLP groups was upregulated compared to the CON, LP, and DLP groups (*p* < 0.05). Moreover, the mRNA expression of the claudin-1 gene of fattening pigs in the DLP + CE group was upregulated compared to that in the FDLP group (*p* < 0.05). There were no significant changes in the mRNA expression levels of occludin among dietary groups (*p* > 0.05).

## 4. Discussion

### 4.1. Nitrogen Balance

Nitrogen is a significant component of pollutants discharged from pig farms, and the amount of nitrogen emissions is closely related to the protein levels in the diets. The total nitrogen emissions from fattening pigs can be decreased by approximately 8% for every 1% dietary CP reduction [14]. The current study showed a 16% reduction in FN and a 25% reduction in TNE in the DLP + CE and FDLP groups compared with the CON group. Similarly, Liu et al. found that the low-protein diet with or without fermentation improved the growth performance and reduced the nitrogen excretion of finishing pigs [15]. Nitrogen deposition refers to the differences between nitrogen intake and total nitrogen excretion, and the amount of nitrogen deposition reflects the efficiency of nitrogen utilization from the diet in pigs. However, this study showed the reduced dietary CP level did not significantly affect nitrogen deposition; it was also found in previous studies that reducing the dietary CP level from 16.5% to 12.5% did not result in a significant difference in nitrogen deposition [16].

### 4.2. Growth Performance

The rational utilization of unconventional feed ingredients is important for the sustainable development of the livestock industry. In this study, LP or DLP diets with or without cellulose supplementation and fermentation did not significantly affect the ADG, ADFI, or feed:gain ratios in fattening pigs. Previous studies have shown that LP diets supplemented with lysine, threonine, methionine, tryptophan, valine, and isoleucine can reduce feed costs without negative effects on the growth performance of fattening pigs [2]. Moreover, the growth performance of pigs was not affected when the dietary CP content was reduced by 2–3% [14], which was consistent with the results of this study. Therefore, by reasonably supplementing essential amino acids, the negative impact of LP on growth performance can be prevented in growing and finishing Landrace × Rongchang pigs. However, studies about LP diets have primarily focused on corn–soybean meal formulations, and there are few studies focusing on using unconventional feeds especially in LP diets for pigs. In this study, the DLP diet had no significant effect on the growth performance of Landrace × Rongchang pigs, indicating that unconventional feeds such as broken rice, rapeseed meal and DDGS could be potentially used in pig diets to replace corn and soybean meal. Unconventional feed resources like DDGS, broken rice, and other agro-industrial byproducts are increasingly used to partially replace conventional ingredients (e.g., soybean meal, corn) in livestock diets, helping to reduce feed costs while utilizing local resources. For instance, a previous study used 100% brown rice to replace corn in the diets of fattening pigs, and found no adverse effects on the growth or slaughter performance of pigs [17]. Additionally, studies reported that 9% rapeseed meal or 20% DDGS in the normal-protein-level diets did not show adverse effects on the growth performance but increased the economic benefits of fattening pigs [18,19]. Overall, compared to the CON diet, the DLP diet with cellulose or fermentation would reduce the feed cost and the reliance on traditional feeds. It demonstrated that broken rice, rapeseed meal, and DDGS can partially replace corn and soybean meal, and exhibited favorable economic benefits without compromising growth performance, which is of great importance for industrial application.

### 4.3. Plasma Biochemical Indices and Jejunal Mucosal Cytokines

In this study, the LP and DLP diets reduced the plasma BUN levels and did not affect the AST levels in pigs. In line with this finding, a previous study found that LP diets tended to lower plasma BUN levels in growing–finishing pigs without affecting plasma AST levels [20]. Similarly, a previous study has shown that unconventional feeds replacing soybean meal could lower plasma BUN levels in growing–finishing pigs [21]. This suggests that LP or DLP diets may improve the amino acid balance and nitrogen metabolism in growing Landrace × Rongchang pigs. Additionally, this study showed that the FDLP and DLP + CE diets reduced plasma BUN levels in growing pigs compared with the CON diet, indicating that fermentation and enzyme supplementation in LP diets might enhance nitrogen absorption and improve protein utilization efficiency.

In this study, the plasma GH concentrations of pigs in the growing phase were reduced with LP and DLP diets. In the fattening phase, the LP and DLP diets did not significantly change the plasma GH levels of pigs, which may be attributed to the higher nutritional demands of growing pigs, whose digestive systems are still developing, making them more sensitive to dietary nutrient changes such as decreased dietary protein intake [22]. Additionally, the FDLP diet increased plasma GH concentrations compared with the LP and DLP diets in fattening Landrace × Rongchang pigs. This enhancement may be due to the cellulase supplementation and microbial fermentation of feed, which convert complex macromolecules such as carbohydrates, proteins, and fats into more readily absorbable molecules, thereby improving the digestibility of nutrients and increasing the supply of amino acids, glucose, and other nutrients [23].

The plasma T-AOC levels of growing pigs were increased with the LP diet than the CON and DLP diets, which suggested that the LP diet might be beneficial for the antioxidant capacity of Landrace × Rongchang pigs during the growing phase. In addition, the DLP diet reduced the plasma T-AOC levels compared with the CON and LP diets during the growing phase, which may be related to the anti-nutritional factors in unconventional feedstuffs. Moreover, we found that the plasma GSH-Px and SOD levels of pigs during the growing phase were increased with FDLP and DLP + CE diets, respectively, compared to the DLP diet. Similar results have been reported in previous studies in which fermented unconventional feed ingredients and exogenous enzyme preparations improved antioxidant capacity in livestock [24,25]. This suggests that feed fermentation or enzyme supplementation positively influence the antioxidant capacity of growing pigs, possibly attributed to the enhanced nutrient utilization from unconventional feed ingredients and the facilitation of antioxidant enzyme and bioactive substance synthesis in animals [26].

### 4.4. Carcass Characteristics and Meat Quality

Carcass characteristics including backfat thickness, carcass weight, and loin eye muscle area are important parameters used to evaluate the carcass quality of pigs. In this study, dietary treatments did not significantly affect the carcass traits of fattening pigs. Similarly, previous study found that adding 15% DDGS to a grain–soybean meal-based diet did not affect the carcass traits of fattening pigs [27]. In addition, it was reported that replacing soybean meal with peas, lentils, and canola oil did not adversely affect the carcass traits or meat quality of animals [28]. Therefore, it demonstrated that unconventional ingredients such as broken rice, rapeseed meal, and DDGS could be potentially used to replace corn and soybean meal in pig diets without negatively impacting the carcass traits of Landrace × Rongchang fattening pigs. The cellulose supplementation improved the marbling score and IMF content of the LD muscle in fattening pigs fed with the DLP diet. This beneficial effect is likely attributed to the capacity of cellulase to break down the poorly digestible nutrients in unconventional feed ingredients into easily digestible molecules, thereby enhancing the nutrient utilization (including fatty acids and fats) and promote the synthesis and deposition of fatty acids in the muscle. However, DLP, DLP + CE and FDLP diets did not significantly change the meat quality of pigs compared with the LP diet. Similar to our results, previous studies reported that supplementation of brown rice or DDGS with enzymes to a basal diet did not show negative impacts on carcass traits and meat quality in growing pigs [16,29]. A recent study also found that reducing dietary protein levels with or without fermented feed supplementation did not affect the carcass traits and meat quality of growing–fattening pigs [30].

The fatty acid composition is also closely related to the nutritional value of meat, and a high content of saturated fatty acids is less prone to oxidation, which helps maintain the meat quality and prolong its shelf life [31]. Some medium-chain fatty acids, such as lauric acid (C12:0) and capric acid (C10:0) can influence the flavor and texture of the meat [4]. In this study, the DLP diet reduced the C12:0 content in the LD muscle of Landrace × Rongchang fattening pigs compared to the LP diet, and did not significantly change the other fatty acid contents, suggesting the reduced dietary protein levels, enzyme supplementation or feed fermentation had limited effects on the fatty acid composition in the LD muscle of pigs.

### 4.5. Nutrient Transport Carrier in the Jejunal Mucosa

The small intestine is the primary site for glucose and amino acid absorption, wherein SLC1A1 is a sodium-dependent transport protein that primarily facilitates the transport of acidic amino acids. The current study found that the mRNA expression of the SLC1A1 gene in the jejunal mucosa of pigs fed the FDLP diet was higher than that of those in the DLP group. This may be due to the fermentation process improving the nutritional value of unconventional feed ingredients, thereby enhancing protein absorption and amino acid utilization [32]. It is also possible that the increase in plasma GH of pigs in the FDLP group was attributed to the changed expression of amino acid transport proteins [33]. Fatty acid transport proteins (FATPs) function in the cell membrane and are critical for maintaining intracellular fatty acid homeostasis, synthesizing fatty acids, and regulating metabolic pathways [34]. In this study, the LP diet increased the mRNA expression of FATP1 in the jejunal mucosa of Landrace × Rongchang fattening pigs, indicating the increased fatty acid transport in the jejunum compared with CON. Compared to the DLP group, the FATP1 and FATP4 gene expression levels in the jejunal mucosa of fattening pigs increased with the FDLP and DLP + CE diets. These findings suggested that cellulase or fermented feed increased the gene expression of intestinal fatty acid transporters, thereby regulating relevant metabolic pathways and enhancing the utilization efficiency of unconventional feed ingredients.

### 4.6. Gene Expression of Lipid Metabolism, Protein Synthesis and Muscle Fiber in the LD Muscle

Lipid metabolism is modulated by key enzymes, including ACC and FASN, and their variations can impact the synthesis rate of fatty acids [35,36]. FABP4 is related to modulating the expression of genes involved in lipid metabolism and facilitates the transport of fatty acids [37]. In this study, the mRNA expressions of FASN in the LD muscle of pigs were upregulated with the DLP + CE and FDLP diets compared with the LP and DLP diets, and the mRNA expression of FABP4 and FASN were upregulated with the FDLP diet compared with the DLP diet. Similar to our results, a previous study found that fermented feed supplementation upregulated FABP4 mRNA expression in fattening pigs [38]. These results corresponded with the improved marbling score and IMF content of the LD muscle fed with the DLP + CE diet, indicating cellulose supplementation might promote the synthesis and transport of fatty acids in the LD muscle. The mTOR pathway is one of the main signaling pathways regulating protein synthesis, wherein mTOR complex 1 can promote protein synthesis by activating eIF4E-binding protein 1 (4E-BP1) and p70S6 kinase 1 (S6K1) [39]. In this study, the FDLP diet increased the mRNA expression of the mTOR and 4E-BP1 genes in the LD muscle of fattening pigs compared with the LP and DLP diets, indicating the fermentation process of the DLP diet could regulate muscle protein synthesis.

The muscle fiber types are primarily characterized by myosin heavy chains (MyHC) I, IIa, IIb, and IIx, wherein the proportions of type I, IIa, and IIx muscle fibers have a positive correlation with the muscle color, tenderness, and fat content of animals; in contrast, type IIb muscle fibers have a negative correlation with meat quality traits [40]. In this study, the mRNA expression levels of MyHC IIa and MyHC I genes in the LD muscle were downregulated in the DLP group compared to the CON group. Moreover, compared to the DLP diet, the FDLP diet increased the mRNA expression levels of MyHC I and MyHC IIa genes in the LD muscle of Landrace × Rongchang pigs. This was also reported by a previous study where an LP diet with fermented feed affected the composition and proportion of muscle fibers and enhanced meat quality by modulating the mRNA expression of muscle fiber genes [31].

### 4.7. Microbiota Composition in the Colon and SCFAs in the Colonic Content

The gut microbes are associated with digestion and nutrient metabolism, immune response, intestinal barrier function and inflammation of the host. In the current study, the colonic Firmicutes were enriched in the FDLP group, which might contribute to the fat accumulation and corresponded with the upregulated expression of FASN and FABP4 genes. The relative abundance of Peptostreptococcaceae, Clostridiaceae, and Clostridium sensu stricto 1 was increased in the colon contents of pigs fed the DLP diet, whereas no increase was observed in pigs fed the FDLP and DLP + CE diets. Some members of the Clostridia class include pathogenic species such as Clostridium perfringens, Clostridium difficile, and Clostridium botulinum [41]. Previous studies have indicated that feeding weaned piglets with fermented feed can decrease the relative abundance of Clostridia in the gut [42]. Thus, fermentation and the addition of cellulase may reverse the negative effects of the DLP diet on the intestinal microbiota community and regulate intestinal health. Liu et al. found that a Lactobacillus-fermented low-protein diet altered the fecal microbiota and metabolites in finishing pigs, which possibly contributed to reduced nitrogen excretion [15]. Additionally, the DLP diet reduced the contents of acetate and propionate in the colonic digesta of Landrace × Rongchang pigs compared with the CON diet, while the FDLP diet increased the contents of acetate, propionate, and valerate compared with the DLP diet. Previous studies found that, compared to a control diet with 10% non-fermented defatted rice bran, the diet with 10% fermented defatted rice bran was found to be more beneficial for SCFA production in the intestines, especially acetate and butyrate [43]. Therefore, the reduced contents of SCFAs and changed microbiota community may be related to the low degradation capacity of unconventional feed ingredients, whereas the fermentation or cellulose supplementation of the DLP diet may release degradable nutrients and contribute to the production of SCFAs, which subsequently inhibit the potential pathogens in the pig intestine. The elevated concentrations of acetate, propionate, and valerate in the FDLP group may be linked with the enriched Firmicutes and changed lipid metabolism of the LD muscle of Landrace × Rongchang pigs. These results indicated a potential link among gut microbiota, SCFA production, and lipid metabolism in the muscle of pigs.

### 4.8. Tight Junction Proteins in the Colonic Mucosa

The intestinal barrier consists of a series of defense mechanisms in the intestinal epithelium that protect the internal environment against pathogens, toxins, and antigens [44]. In this study, the LP and DLP diets did not change the mRNA expression of claudin-1, ZO-1, and occludin in the colonic mucosa of pigs compared with the CON diet. However, the DLP + CE and FDLP diets increased claudin-1 mRNA expression in the colon mucosa, and the FDLP diet upregulated ZO-1 expression. Similarly, a previous study reported that diets with fermented feed increased occludin, ZO-1, and claudin-1 mRNA expression in the jejunum mucosa of weaned piglets [42]. Therefore, it indicated that cellulose or fermented feed supplementation improved intestinal barrier functions in pigs.

## 5. Conclusions

The partial replacement of corn and soybean meal with unconventional feed ingredients (broken rice, rapeseed meal, and DDGS) in LP diets did not show negative effects on growth performance, meat quality, and carcass traits of Landrace × Rongchang fattening pigs. Furthermore, the dietary addition of cellulase or 20% FDLP can improve nitrogen utilization and intestinal barrier functions in Landrace × Rongchang pigs, indicating that the utilization efficiency of LP diets based on diversified feed ingredients can be improved by using cellulose supplementation and microbial fermentation, which is beneficial for reducing environmental pollution and supporting sustainable livestock production.

## Figures and Tables

**Table 1:** Composition and nutrient levels of diets in the growing period (air-dried basis, %).

Items	Treatment				
	CON	LP	DLP	DLP + CE	FDLP
Ingredients					
Corn	64.58	69.28	53.34	53.34	41.91
Soybean meal	16.80	12.18	2.98	2.98	2.38
FDLP	0.00	0.00	0.00	0.00	20.00
Broken rice	0.00	0.00	11.44	11.39	9.15
Rapeseed meal	0.00	0.00	6.90	6.90	5.52
DDGS	0.00	0.00	8.80	8.80	7.04
Wheat bran	14.54	14.53	11.90	11.90	9.52
Soybean oil	0.55	0.20	0.80	0.80	0.64
L-Lysine hydrochloride (78.8%)	0.42	0.54	0.66	0.66	0.66
DL-methionine (99%)	0.06	0.08	0.05	0.05	0.05
L-threonine (98.5%)	0.10	0.16	0.19	0.19	0.19
L-tryptophan (98%)	0.01	0.04	0.05	0.05	0.05
Limestone	0.65	0.69	0.75	0.75	0.75
CaHPO_4_·2H_2_O	0.90	0.91	0.75	0.75	0.75
NaCl	0.30	0.30	0.30	0.30	0.30
Antioxidants	0.03	0.03	0.03	0.03	0.03
Fungicide	0.06	0.06	0.06	0.06	0.06
Cellulase	0.00	0.00	0.00	0.05	0.00
Premix ^1^	1.00	1.00	1.00	1.00	1.00
Total	100.00	100.00	100.00	100.00	100.00
Nutrient level (%)					
Net energy (Mcal/kg) ^2^	2.34	2.34	2.34	2.34	2.34
CP ^3^	15.20	13.66	13.49	13.49	13.81
ADF ^3^	5.20	5.03	6.27	6.27	6.06
NDF ^3^	13.77	13.65	14.54	14.54	14.36
Ca ^2^	0.60	0.60	0.60	0.60	0.60
P ^2^	0.28	0.28	0.28	0.28	0.28
SID lysine ^4^	0.87	0.87	0.87	0.87	0.87
SID methionine ^4^	0.26	0.26	0.26	0.26	0.26
SID threonine ^4^	0.54	0.54	0.54	0.54	0.54
SID tryptophan ^4^	0.15	0.15	0.15	0.15	0.15

^1^ Providing the following per kg diet: Cu (as copper sulfate), 100 mg; Fe (as ferrous sulfate), 100 mg; Zn (as zinc oxide), 120 mg; Mn (as manganese sulfate), 20 mg; I (as calcium iodate), 0.3 mg; and Se (as sodium selenite), 0.3 mg; vitamin A, 3, 800 IU; vitamin D3 or 25-hydroxyvitamin D 3, 800 IU; vitamin E, 10 IU; vitamin K, 1 mg; choline, 200 mg; pantothenic, 5 mg; vitamin B2, 2 mg; folic acid, 0.8 mg; vitamin B1, 1 mg; vitamin B6, 1 mg; biotin, 0.08 mg; vitamin B12, 0.01 mg. ^2^ Calculated values. ^3^ Measured values. ^4^ Values for the SID AAs were calculated using standardized ileal digestible coefficients for the various ingredients provided by NRC (2012). CON, control diet; LP, low-protein diet; DLP, diversified low-protein diet; DLP + CE, diversified low-protein diet with cellulase; FDLP, diversified low-protein diet with fermented feed; CP, crude protein; ADF, acid detergent fiber; NDF, neutral detergent fiber; Ca, calcium; P, phosphorus; SID, standardized ileal digestible.

**Table 2:** Composition and nutrient levels of diets in fattening period (air-dried basis, %).

Items	Treatment				
	CON	LP	DLP	DLP + CE	FDLP
Ingredients					
Corn	71.11	74.34	58.09	58.09	45.77
Soybean meal	10.70	5.40	0.00	0.00	0.00
FDLP	0.00	0.00	0.00	0.00	20.00
Broken rice	0.00	0.00	14.73	14.68	11.78
Rapeseed meal	0.00	0.00	4.45	4.45	3.56
DDGS	0.00	0.00	5.20	5.20	4.16
Wheat bran	14.59	16.44	13.68	13.68	10.94
Soybean oil	0.30	0.25	0.30	0.30	0.24
L-Lysine hydrochloride (78.8%)	0.40	0.52	0.59	0.59	0.59
DL-methionine (99%)	0.03	0.05	0.03	0.03	0.03
L-threonine (98.5%)	0.10	0.17	0.18	0.18	0.18
L-tryptophan (98%)	0.02	0.05	0.06	0.06	0.06
Limestone	0.64	0.68	0.78	0.78	0.78
CaHPO_4_·2H_2_O	0.72	0.71	0.52	0.52	0.52
NaCl	0.30	0.30	0.30	0.30	0.30
Antioxidants	0.03	0.03	0.03	0.03	0.03
Fungicide	0.06	0.06	0.06	0.06	0.06
Cellulase	0.00	0.00	0.00	0.05	0.00
Premix ^1^	1.00	1.00	1.00	1.00	1.00
Total	100.00	100.00	100.00	100.00	100.00
Nutrient level (%)					
Net energy (Mcal/kg) ^2^	2.37	2.37	2.37	2.37	2.37
CP ^3^	12.91	11.38	11.14	11.15	11.48
ADF ^3^	5.18	5.18	6.72	6.72	6.45
NDF ^3^	13.34	13.54	15.02	15.02	14.78
Ca ^2^	0.52	0.52	0.52	0.52	0.52
P ^2^	0.24	0.24	0.24	0.24	0.24
SID lysine ^4^	0.73	0.73	0.73	0.73	0.73
SID methionine ^4^	0.21	0.21	0.21	0.21	0.21
SID threonine ^4^	0.46	0.46	0.46	0.46	0.46
SID tryptophan ^4^	0.13	0.13	0.13	0.13	0.13

^1^ Providing the following per kg diet: Cu (as copper sulfate), 100 mg; Fe (as ferrous sulfate), 100 mg; Zn (as zinc oxide), 120 mg; Mn (as manganese sulfate), 20 mg; I (as calcium iodate), 0.3 mg; and Se (as sodium selenite), 0.3 mg; vitamin A, 3, 800 IU; vitamin D3 or 25-hydroxyvitamin D 3, 800 IU; vitamin E, 10 IU; vitamin K, 1 mg; choline, 200 mg; pantothenic, 5 mg; vitamin B2, 2 mg; folic acid, 0.8 mg; vitamin B1, 1 mg; vitamin B6, 1 mg; biotin, 0.08 mg; vitamin B12, 0.01 mg. ^2^ Calculated values. ^3^ Measured values. Values for SID AAs were calculated using standardized ileal digestible coefficients for the various ingredients provided by NRC (2012). CON, control diet; LP, low-protein diet; DLP, diversified low-protein diet; DLP + CE, diversified low-protein diet with cellulase; FDLP, diversified low-protein diet with fermented feed; CP, crude protein; ADF, acid detergent fiber; NDF, neutral detergent fiber; Ca, calcium; P, phosphorus; SID, standardized ileal digestible. ^4^ Standard Ileal digestible amino acids.

**Table 3:** Effects of diversified low-protein diet supplemented with enzyme preparation or fermented feed on the nitrogen balance of Landrace × Rongchang growing pigs.

Items	Treatment					SEM	*p*-Value
	CON	LP	DLP	DLP + CE	FDLP		
ADFI (g/d)	1813	1813	1811	1811	1811	143	0.993
IN (g/d)	43.92 ^a^	38.73 ^b^	38.30 ^b^	38.32 ^b^	38.81 ^b^	1.05	<0.001
FN (g/d)	8.44 ^a^	7.42 ^b^	7.69 ^ab^	7.07 ^b^	7.08 ^b^	0.36	0.007
UN (g/d)	11.90 ^a^	8.76 ^b^	9.04 ^b^	8.47 ^b^	8.18 ^b^	0.67	<0.001
TNE (g/d)	20.33 ^a^	16.21 ^b^	16.74 ^b^	15.54 ^b^	15.31 ^b^	0.85	<0.001
RN (g/d)	23.11	20.30	19.82	20.92	21.53	1.16	0.076
RN/IN (%)	52.62	52.43	51.70	54.52	55.43	2.30	0.164

^a,b^ Means in the same row with different superscripts differ (*p* < 0.05). Values are means and standard error of the means (*n* = 5). ADFI, average daily feed intake; IN, intake of nitrogen; FN, fecal nitrogen; UN, urine nitrogen; TNE, total nitrogen excretion; RN, retained nitrogen; RN/IN, the ratio of nitrogen retention to nitrogen intake; CON, control diet; LP, low-protein diet; DLP, diversified low-protein diet; DLP + CE, diversified low-protein diet with cellulase; FDLP, diversified low-protein diet with fermented feed.

**Table 4:** Effects of diversified low-protein diet supplemented with enzyme preparation or fermented feed on growth performance of Landrace × Rongchang growing–finishing pigs.

Items	Treatments					SEM	*p*-Value
	CON	LP	DLP	DLP + CE	FDLP		
Days 1 to 47 (30–60 kg)							
Initial body weight (kg)	31.10	31.31	31.02	31.13	31.10	0.22	0.684
Final body weight (kg)	62.52	62.44	61.30	65.91	65.32	1.79	0.061
ADG (kg)	0.77	0.76	0.74	0.85	0.83	0.04	0.063
ADFI (kg)	2.10	2.03	2.11	2.21	2.24	0.07	0.062
F/G	2.75	2.71	2.88	2.61	2.69	0.12	0.310
Days 47 to 86 (60–100 kg)							
Initial body weight (kg)	62.52	62.44	61.30	65.91	65.32	1.79	0.060
Final body weight (kg)	93.60	94.81	92.53	94.41	95.22	0.66	0.733
ADG (kg)	0.80	0.83	0.80	0.73	0.76	0.02	0.434
ADFI (kg)	2.85	2.91	2.72	2.62	2.46	0.06	0.102
F/G	3.59	3.52	3.40	3.59	3.22	0.07	0.183

Values are presented as means and standard error of the means (*n* = 6). ADG, average daily gain; ADFI, average daily feed intake; F/G, feed to gain ratio. CON, control diet; LP, low-protein diet; DLP, diversified low-protein diet; DLP + CE, diversified low-protein diet with cellulase; FDLP, diversified low-protein diet with fermented feed.

**Table 5:** Effects of diversified low-protein diet supplemented with enzyme preparation or fermented feed on plasma biochemical indices of Landrace × Rongchang growing–finishing pigs.

Items	Treatment					SEM	*p*-Value
	CON	LP	DLP	DLP + CE	FDLP		
Days 1 to 47 (30–60 kg)							
BUN (mmol/L)	2.87 ^a^	1.82 ^b^	1.82 ^b^	1.53 ^b^	1.64 ^b^	0.28	0.001
AST (U/L)	2.23	2.81	3.94	3.39	2.86	0.80	0.293
GH (ng/mL)	20.14 ^a^	13.43 ^b^	11.01 ^b^	15.81 ^ab^	12.72 ^b^	2.44	0.012
INS (pg/mL)	189	198	180	193	210	22	0.750
CAT(U/mL)	3.75	3.09	3.41	4.87	2.12	1.16	0.244
T-AOC (Mm)	0.27 ^b^	0.32 ^a^	0.24 ^c^	0.25 ^bc^	0.27 ^b^	0.01	0.001
SOD (U/mL)	13.94 ^b^	12.54 ^b^	12.83 ^b^	15.44 ^a^	12.92 ^b^	0.71	0.002
MDA (nmol/mL)	4.29 ^ab^	4.08 ^b^	5.20 ^a^	3.60 ^b^	3.25 ^b^	0.52	0.011
GSH-Px (U/mL)	425 ^b^	433 ^b^	412 ^b^	503 ^ab^	707 ^a^	101	0.042
IgM (mg/mL)	1.57	1.89	1.75	2.68	3.11	0.60	0.070
IgA (µg/mL)	119	121	115	120	126	22	0.990
IgG (mg/mL)	0.80	0.87	0.76	0.92	0.90	0.26	0.971
Days 47 to 86 (60–100 kg)							
BUN (mmol/L)	1.65	1.55	1.54	1.58	1.59	0.11	0.873
AST (U/L)	5.02	5.17	5.98	3.74	4.99	1.24	0.525
GH (ng/mL)	15.90 ^ab^	12.92 ^b^	12.52 ^b^	16.33 ^ab^	18.40 ^a^	1.70	0.013
INS (pg/mL)	95.93	97.63	93.12	96.14	98.64	12.5	0.991
CAT (U/mL)	7.13	7.74	7.14	7.03	8.28	1.64	0.930
T-AOC (Mm)	0.25	0.25	0.22	0.23	0.24	0.02	0.582
SOD (U/mL)	14.61	15.83	16.50	16.33	15.60	0.85	0.232
MDA (nmol/mL)	3.96	4.25	4.50	4.13	4.38	0.81	0.971
GSH-Px (U/mL)	834	862	845	874	887	41	0.713
IgM (mg/mL)	2.85	2.91	2.28	3.07	2.81	0.76	0.875
IgA (µg/mL)	383	391	373	385	392	35	0.984
IgG (mg/mL)	1.55	1.60	1.52	1.60	1.64	0.24	0.992

^a,b,c^ Values within a row followed by different superscript letters are significantly different at *p* < 0.05. Values are showed as means and standard error of the means (*n* = 6). BUN, blood urea nitrogen; AST, aspartate aminotransferase; GH, growth hormone; INS, insulin; CAT, catalase; T-AOC, total antioxidant capacity; SOD, superoxide dismutase; MDA, malondialdehyde; GSH-Px, glutathione peroxidase; IgM, immunoglobulin M; IgA, immunoglobulin A; IgG, immunoglobulin G; CON, control diet; LP, low-protein diet; DLP, diversified low-protein diet; DLP + CE, diversified low-protein diet with cellulase; FDLP, diversified low-protein diet with fermented feed.

**Table 6:** Effects of diversified low-protein diet supplemented with enzyme preparation or on carcass traits and meat quality of Landrace × Rongchang finishing pigs.

Items	Treatment					SEM	*p*-Value
	CON	LP	DLP	DLP + CE	FDLP		
Carcass weight (kg)	77.62	76.01	75.91	78.02	76.81	1.63	0.643
Carcass yield (%)	72.44	71.91	72.04	72.50	72.52	0.61	0.832
Carcass straight length (cm)	95.70	99.20	94.52	97.31	98.52	2.33	0.274
Carcass skew length (cm)	80.72	83.31	79.51	80.22	81.83	2.18	0.454
Loin eye area (cm^2^)	32.64	26.94	27.73	26.72	26.70	2.41	0.091
Back fat thickness at the thickest part of the shoulder (mm)	50.83	51.62	54.44	55.52	54.32	3.32	0.574
Back fat thickness at thoracolumbar junction (mm)	29.12	24.80	32.81	28.82	27.13	2.87	0.102
Back fat thickness at lumbar–sacral junction (mm)	30.60	29.32	32.14	33.24	33.33	2.62	0.493
Back fat thickness at the joint of 6~7 ribs (mm)	3.52	4.08	4.04	4.01	4.16	0.31	0.282
pH 45 min	6.33	6.15	6.24	6.21	6.31	0.97	0.353
pH 24 h	5.43 ^b^	5.52 ^ab^	5.48 ^b^	5.43 ^b^	5.65 ^a^	0.07	0.033
Marbling Score	2.50 ^bc^	2.92 ^abc^	2.33 ^c^	3.25 ^a^	3.00 ^ab^	0.29	0.031
Colorimetric card rating	3.50	3.50	3.67	3.50	3.50	0.07	0.072
Color measurement L*	43.51	44.01	41.84	43.93	43.62	1.09	0.270
Color measurement a*	5.24	4.89	5.23	5.74	5.04	0.75	0.830
Color measurement b*	2.35	2.17	1.80	2.45	2.47	0.37	0.382
Drip loss (%)	2.60	2.88	3.07	3.30	2.71	0.55	0.721
Cooking loss (%)	31.95	31.64	32.34	30.30	29.61	1.39	0.281
Shear force (N)	37.33	37.41	37.01	36.23	36.12	2.19	0.953
Inosine acid (%)	3.08	3.07	2.95	3.12	3.08	0.15	0.951
Intramuscular fat (%)	3.70 ^b^	4.20 ^ab^	3.18 ^b^	5.64 ^a^	4.17 ^ab^	0.72	0.034

^a,b,c^ Values within a row followed by different superscript letters are significantly different at *p* < 0.05. Values are showed as means and standard error of the means (*n* = 6). CON, control diet; LP, low-protein diet; DLP, diversified low-protein diet; DLP + CE, diversified low-protein diet with cellulase; FDLP, diversified low-protein diet with fermented feed.

**Table 7:** Effects of diversified low-protein diet supplemented with enzyme preparation or fermented feed on long-chain fatty acids in longissimus dorsi muscle of Landrace × Rongchang finishing pigs.

Items (%)	Treatment					SEM	*p*-Value
	CON	LP	DLP	DLP + CE	FDLP		
Methyl decanoate (C10:0)	0.093	0.104	0.085	0.102	0.093	0.005	0.053
Methyl laurate (C12:0)	0.085 ^ab^	0.094 ^a^	0.073 ^b^	0.083 ^ab^	0.085 ^ab^	0.004	0.032
Methyl tetradecanoate (C14:0)	1.36	1.32	1.27	1.34	1.35	0.97	0.902
Methyl palmitate (C16:0)	27.45	27.15	27.07	28.15	27.23	0.63	0.453
Methyl palmitoleate (C16:1)	3.11	3.03	2.80	3.31	2.95	0.26	0.421
Methyl heptadecanoate (C17:0)	0.16	0.17	0.15	0.15	0.15	0.01	0.503
Methyl octadecenoate (C18:0)	15.04	15.22	15.12	15.33	15.54	0.60	0.931
trans-9-Elaidic acid methyl ester (C18:1n9t)	0.133	0.135	0.134	0.133	0.132	0.005	0.892
cis-9-0leic acid methyl ester (C18:1n9c)	38.14	38.74	38.93	39.02	38.22	1.89	0.990
Methyl Linoleate (C18:2n6c)	9.82	9.21	9.83	8.58	9.71	1.41	0.884
Methyl Arachidate (C20:0)	0.26	0.29	0.27	0.28	0.28	0.28	0.874
Methyl cis-11-eicosenoate (C20:1)	0.77	0.80	0.84	0.81	0.85	0.09	0.932
Methyl Linolenate (C18:3n3)	0.28	0.25	0.28	0.26	0.27	0.02	0.345
cis-11,14-Eicosatrienoic acid methyl ester (C20:2)	0.34	0.59	0.34	0.28	0.32	0.12	0.132
cis-8,11,14-Eicosatrienoic acid methyl ester (C20:3n6)	0.29	0.32	0.27	0.25	0.27	0.07	0.875
Methyl tricosanoate (C20:4n6)	2.24	2.10	2.01	1.74	2.05	0.59	0.942
Methyl cis-5,8,11,14-Eicosatetraenoic (C23:0)	0.50	0.55	0.53	0.50	0.58	0.15	0.980

^a,b^ Values within a row followed by different superscript letters are significantly different at *p* < 0.05. Values are showed as means and standard error of the means (*n* = 6). CON, control diet; LP, low-protein diet; DLP, diversified low-protein diet; DLP + CE, diversified low-protein diet with cellulase; FDLP, diversified low-protein diet with fermented feed.

**Table 8:** Effects of diversified low-protein diet supplemented with enzyme preparation or fermented feed on SCFA content in colonic contents of Landrace × Rongchang finishing pigs.

Items (%)	Treatment					SEM	*p*-Value
	CON	LP	DLP	DLP + CE	FDLP		
Acetic acid (μg/g)	1387 ^a^	1299 ^ab^	1029 ^b^	1259 ^ab^	1458 ^a^	130	0.034
Propanoic acid (μg/g)	725 ^a^	685 ^a^	508 ^b^	631 ^ab^	784 ^a^	69	0.012
Isobutyric acid (μg/g)	78.74	72.52	62.72	68.73	77.64	7.04	0.172
Butyric acid (μg/g)	294	286	232	343	363	56	0.183
Isovaleric acid (μg/g)	135	147	104	123	122	17	0.171
Valeric acid (μg/g)	84.31 ^ab^	68.73 ^b^	64.74 ^b^	99.54 ^a^	101.02 ^a^	12.73	0.022

^a,b^ Values within a row followed by different superscript letters are significantly different at *p* < 0.05. Values are shown as means and standard error of the means (*n* = 6). CON, control diet; LP, low-protein diet; DLP, diversified low-protein diet; DLP + CE, diversified low-protein diet with cellulase; FDLP, diversified low-protein diet with fermented feed.

## Data Availability

The original contributions presented in this study are included in the article/Supplementary Materials. Further inquiries can be directed to the corresponding authors.

## Supplementary Materials

The following supporting information can be downloaded at: https://www.mdpi.com/article/10.3390/vetsci13030219/s1, Table S1: Primers for real-time PCR in this study; Table S2: Effects of diversified low-protein diets supplemented with enzyme preparations or fermented feed on the colonic microbiota community structure at the phylum level in Landrace × Rongchang finishing pigs; Table S3: Effects of diversified low-protein diets supplemented with enzyme preparations or fermented feed on the colonic microbiota community structure at the family level in Landrace × Rongchang finishing pigs; Table S4: Effects of diversified low-protein diets supplemented with enzyme preparations or fermented feed on the colonic microbiota community structure at the genus level in Landrace × Rongchang finishing pigs; Figure S1: Effects of diversified low-protein diet supplemented with enzyme preparation or fermented feed on colon microbial diversity of Landrace × Rongchang finishing pigs (*n* = 6); Figure S2: The relative abundances of bacteria at the phylum, family, and genus levels (*n* = 6).

## Abbreviations

The following abbreviations are used in this manuscript:

ACC	Acetyl-CoA carboxylase
ADG	Average daily gain
ADFI	Average daily feed intake
ANOVA	Analysis of variance
ASVs	Amplicon Sequence Variants
BUN	Blood urea nitrogen
CAT	Catalase
CE	Cellulose
DDGS	Distillers dried grains with solubles
ELISA	Enzyme-linked immunosorbent assay
FATP	Fatty acid transport protein
FN	Fecal nitrogen
GH	Growth hormone
GLUT2	Glucose transporter 2
GOT	Aspartate aminotransferase
GSH-Px	Glutathione peroxidase
LD	Longissimus dorsi
LP	Low protein
HSL	Hormone-sensitive triglyceride lipase
IgA	Immunoglobin A
IgG	Immunoglobin G
IgM	Immunoglobin M
IL	Interleukin
IN	Nitrogen intake
INS	Insulin
MDA	Malondialdehyde
RN	Retained nitrogen
SCFAs	Short-chain fatty acids
SGLT1	Sodium-glucose co-transporter 1
SLC1A5	Alanine-serine-cysteine transporter 2
SLC7A1	Cationic amino acid transporter 1
SLC7A7	Solute carrier family 7 member 7
T-AOC	Total antioxidant capacity
TGF-β1	Transforming growth factor-β1
TNE	Total nitrogen excretion
TNF-α	Tumor necrosis factor-α
T-SOD	Total superoxide dismutase
UN	Urinary nitrogen
UPGMA	Unweighted Pair-group Method with Arithmetic Means
ZO-1	Zonula occludens protein-1
