# Supplementary material for: Effects of Enzymes or Fermented Feed on Nitrogen Balance, Meat Quality, Intestinal Microbiota Profile and Barrier Functions of Landrace × Rongchang Pigs Fed with a Diversified Low-Protein Diet
Source: Vet Sci. 2026 Feb 26;13(3):219. doi: 10.3390/vetsci13030219 (PMC13030152; doi:10.3390/vetsci13030219)
Supplement: Supplementary file 1 [file vetsci-13-00219-s001.zip › vetsci-4147994-supplementary.pdf]

Table S1. Primers for real-time PCR in this study

Gene[1]	5′→3′ sequence	Length (bp)	Tm (℃)
GAPDH	F: ACATCATCCCTGCTTCTACTGG<br>R: ATGCCTGCTTCACCACC	183	60
MyHC I	F: GTTTGCCAACTATGCTGGGG<br>R: TGTGCAGAGCTGACACAGTC	95	60
MyHC IIa	F: CTCTGAGTTCAGCAGCCATGA<br>R: GATGTCTTGGCATCAAAGGGC	83	60
MyHC IIb	F: CAGTGAAAGAAGACCAGGTGTTCCC<br>R: GTGTAGATCATCCAGGCTGCGTAAC	139	60
MyHC IIx	F: CAGTGAAAGAAGACCAGGTGTTCCC<br>R: GTGTAGATCATCCAGGCTGCGTAAC	139	60
ACC	F: CAACAATGGCATCGCAGCAGTG<br>R: GGCTTTCAGGTCTTCGGGTGTG	121	60
FASN	F: CCACTCCAAGCAGGCGAACAC<br>R: CACGAAGGGAAGCAGGGTTGATG	104	60
FABP4	F: ACAGGAAAGTCAAGAGCACCATAACC<br>R:<br>GTACATTCCACCACCAACTTATCATCTAC	125	60
HSL	F: CACAAGGGCTGCTTCTACGG R:<br>AAGCGGCCACTGGTGAAGAG	167	60
mTOR	F: ATGTAAACGAAGCCTTAGTGG<br>R: CCGACCGATGTCTGTGAG	146	58.8
4E-BP1	F: CACCTGTGACCAAAACGCCC R:<br>TTGTCGTCTGGGCTACTGCG	121	60
ZO-1	F: GTCAACCCACCAAACCCACCAAAG<br>R: TGCCATCTCTTGCTGCCAAACTATC	129	60
Occludin	F: GTAGTCGGGTTCGTTTCC<br>R: GACCTGATTGCCTAGAGTGT	85	60
Claudin-1	F: GATTTACTCCTACGCTGGTGAC<br>R: CACAAAGATGGCTATTAGTCCC	83	60
SGLT1	F: TGTATTTGAGGCCAGTGTCA<br>R: GGGCGACCACAACTCTTAAA	198	60
GLUT2	F: TGGAATCAGCCAACCTGTTT<br>R: ACAAGTCCCACCGACATGA	165	60
SGLT3	F: TTGTAGCTGCGTGGTACTGG<br>R: ACCATGAGGAACATGGGCAG	165	60
SLC7A1	F: GCCTGAGAGCAAGACCAAAC<br>R: GCCGTAGCCGAAGTAGATGA	166	60
SLC7A7	F: TTTGGTTCCCAAGGTTGCA<br>R: GCAGCTTCCTGGCATTGC	95	60
SLC1A1	F: TCCACTCCATTGTTATTCTGCC<br>R: TTGTCCACCTGGTTCTTCTCTTC	172	60
SLC1A5	F: AAGGAGTCGGTTCTGTGATGG<br>R: TAAAAGTCGGCGAGGGTGA	108	60
FATP1	F: GGCAACAGACGTGATCTATGAC<br>R: AGCGGCTGGCTGAAAACT	125	60
FATP4	F: AGCCGCATCCTGTCCTTT<br>R: GACATCCTTGGCGATCTTTT	213	60
β-actin	F: TGGAACGGTGAAGGTGACAGC<br>R: GCTTTTGGGAAGGCAGGGACT	177	60

GAPDH, glyceraldehyde-3-phosphate dehydrogenase; *MyHC Ⅰ*, myosin heavy chain 1; *MyHC Ⅱa*, myosin heavy chain Ⅱa; *MyHC Ⅱb*, myosin heavy chain Ⅱb; *MyHC Ⅱx*, myosin heavy chain Ⅱx; *ACC*, acetyl-CoA carboxylase; *FASN*, fatty acid synthase; *FABP4,* fatty acid binding protein 4; *HSL,* hormone-sensitive triglyceride lipase; *mTOR*, mammalian target of rapamycin; *4E-BP1*, eukaryotic translation initiation factor 4E (eIF4E)-binding protein 1; *ZO-1,* zonula occludens protein 1; *SGLT1*, sodium-glucose linked transporter 1; *GLUT2*, glucose transporter type 2; *SGLT3*, sodium-glucose linked transporter 3; *SLC7A1*, solute carrier family 7 member 1; *SLC7A7*, solute carrier family 7 member 7; *SLC1A1*, solute carrier family 1 member 1; *SLC1A5*, solute carrier family 1 member5; *FATP1,* fatty acid transport protein 1; *FATP4*, fatty acid transport protein 4.

Table S2. Effects of diversified low-protein diets supplemented with enzyme preparations or fermented feed on the colonic microbiota community structure at the phylum level in *Landrace×Rongchang* finishing pigs

Items(%)	Treatment					SEM	p-value
	CON	LP	DLP	DLP+CE	FDLP		
Firmicutes	0.75^{abc}	0.64^{c}	0.77^{ab}	0.72^{bc}	0.85^{a}	0.051	0.013
Bacteroidetes	0.17^{b}	0.18^{a}	0.11^{ab}	0.17^{ab}	0.071^{b}	0.032	0.007
Spirochaetota	0.038	0.144	0.078	0.064	0.043	0.042	0.120
Actinobacteriota	0.012	0.009	0.022	0.026	0.022	0.007	0.136

^{a,b,c} Means in the same row with different superscripts differ ($p < 0.05$). Values are means and standard error of the means (n=6).

CON, control diet; LP, low-protein diet; DLP, diversified low-protein diet; DLP+CE, diversified low-protein diet with cellulase; FDLP, diversified low-protein diet with biological fermentation.

Table S3. Effects of diversified low-protein diets supplemented with enzyme preparations or fermented feed on the colonic microbiota community structure at the family level in *Landrace×Rongchang* finishing pigs

Items (%)	Treatment					SEM	p-value
	CON	LP	DLP	DLP+CE	FDLP		
Lachnospiraceae	0.169	0.119	0.165	0.146	0.114	0.028	0.176
Oscillospiraceae	0.114	0.113	0.109	0.112	0.101	0.022	0.976
Lactobacillaceae	0.039	0.075	0.082	0.034	0.148	0.046	0.131
Spirochaetaceae	0.038	0.144	0.043	0.064	0.078	0.042	0.120
Muribaculaceae	0.091	0.087	0.040	0.092	0.048	0.034	0.362
Streptococcaceae	0.079	0.032	0.018	0.084	0.057	0.038	0.370
Christensenellaceae	0.049	0.041	0.042	0.046	0.043	0.018	0.988
Peptostreptococcaceae	0.026^{b}	0.031^{b}	0.063^{a}	0.041^{ab}	0.042^{ab}	0.012	0.037
Clostridiaceae	0.017^{b}	0.038^{b}	0.074^{a}	0.034^{b}	0.040^{b}	0.013	0.005
Ruminococcaceae	0.041	0.028	0.046	0.032	0.032	0.008	0.145
norank_o__Clostridia_UCG-014	0.038	0.017	0.044	0.031	0.039	0.013	0.297

^{a,b,c} Means in the same row with different superscripts differ ($p < 0.05$). Values are means and standard error of the means (n=6).

CON, control diet; LP, low-protein diet; DLP, diversified low-protein diet; DLP+CE, diversified low-protein diet with cellulase; FDLP, diversified low-protein diet with fermented feed.

Table S4. Effects of diversified low-protein diets supplemented with enzyme preparations or fermented feed on the colonic microbiota community structure at the genus level in *Landrace×Rongchang* finishing pigs

Items (%)	Treatment					SEM	p-value
	CON	LP	DLP	DLP+CE	FDLP		
Lactobacillus	0.039	0.075	0.148	0.034	0.082	0.046	0.131
Treponema	0.036	0.142	0.077	0.062	0.042	0.040	0.115
norank_f__Muribaculaceae	0.091	0.087	0.048	0.092	0.040	0.034	0.363
unclassified_f__Lachnospiraceae	0.071	0.052	0.047	0.066	0.060	0.016	0.563
Streptococcus	0.079	0.032	0.057	0.084	0.018	0.013	0.370
Christensenellaceae_R-7_group	0.049	0.040	0.042	0.046	0.042	0.017	0.987
UCG-005	0.048	0.053	0.043	0.032	0.042	0.011	0.345
Clostridium_sensu_stricto_1	0.015[b]	0.035[b]	0.073[a]	0.033[b]	0.038[b]	0.012	0.002
norank_f__norank_o__Clostridia_UCG-014	0.038	0.017	0.039	0.031	0.044	0.013	0.297
norank_f__Eubacterium_coprostanoligenes_group	0.044	0.025	0.021	0.036	0.043	0.009	0.091
UCG-002	0.027	0.025	0.025	0.039	0.033	0.015	0.851
Terrisporobacter	0.018	0.020	0.026	0.029	0.041	0.009	0.119
Lachnospiraceae_XPB1014_group	0.033	0.023	0.019	0.027	0.029	0.009	0.652
NK4A214_group	0.025	0.021	0.023	0.025	0.019	0.008	0.947

[a,b,c] Means in the same row with different superscripts differ ($p < 0.05$). Values are means and standard error of the means (n=6).

CON, control diet; LP, low-protein diet; DLP, diversified low-protein diet; DLP+CE, diversified low-protein diet with cellulase; FDLP, diversified low-protein diet with fermented feed.

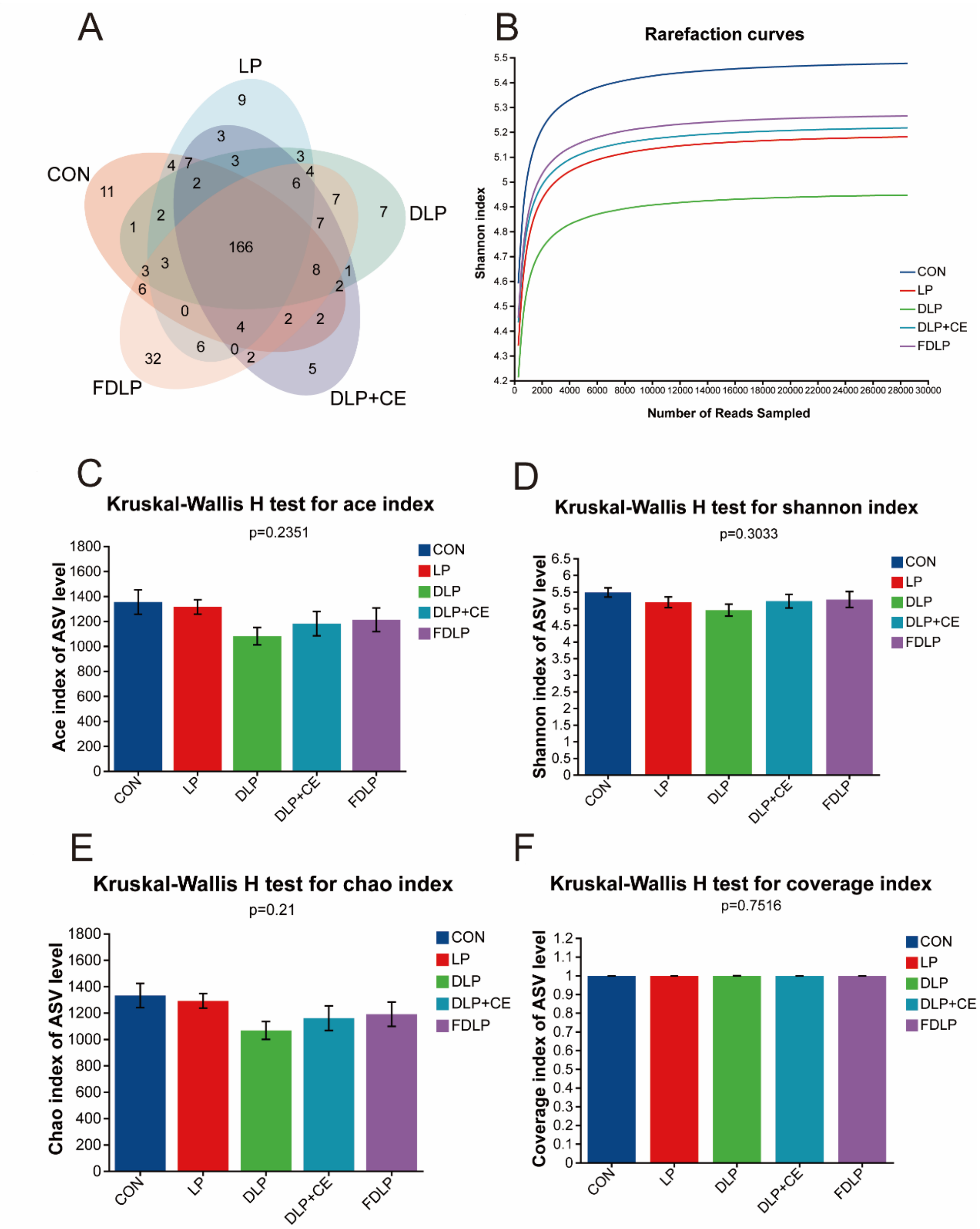

Figure S1 Effects of diversified low-protein diet supplemented with enzyme preparation or fermented feed on colon microbial diversity of *Landrace* × *Rongchang* finishing pigs (n = 6). (**A**) Venn diagram. (**B**) Shannon index. (**C**) Ace index of ASV level. (**D**) Shannon index of ASV level. (**E**) Chao1 index of ASV level. (**F**) Coverage index of ASV level. Abbreviations: CON, control diet; LP, low-protein diet; DLP, diversified low-protein diet; DLP + CE, diversified low-protein diet with cellulase; FDLP, diversified low-protein diet with fermented feed.

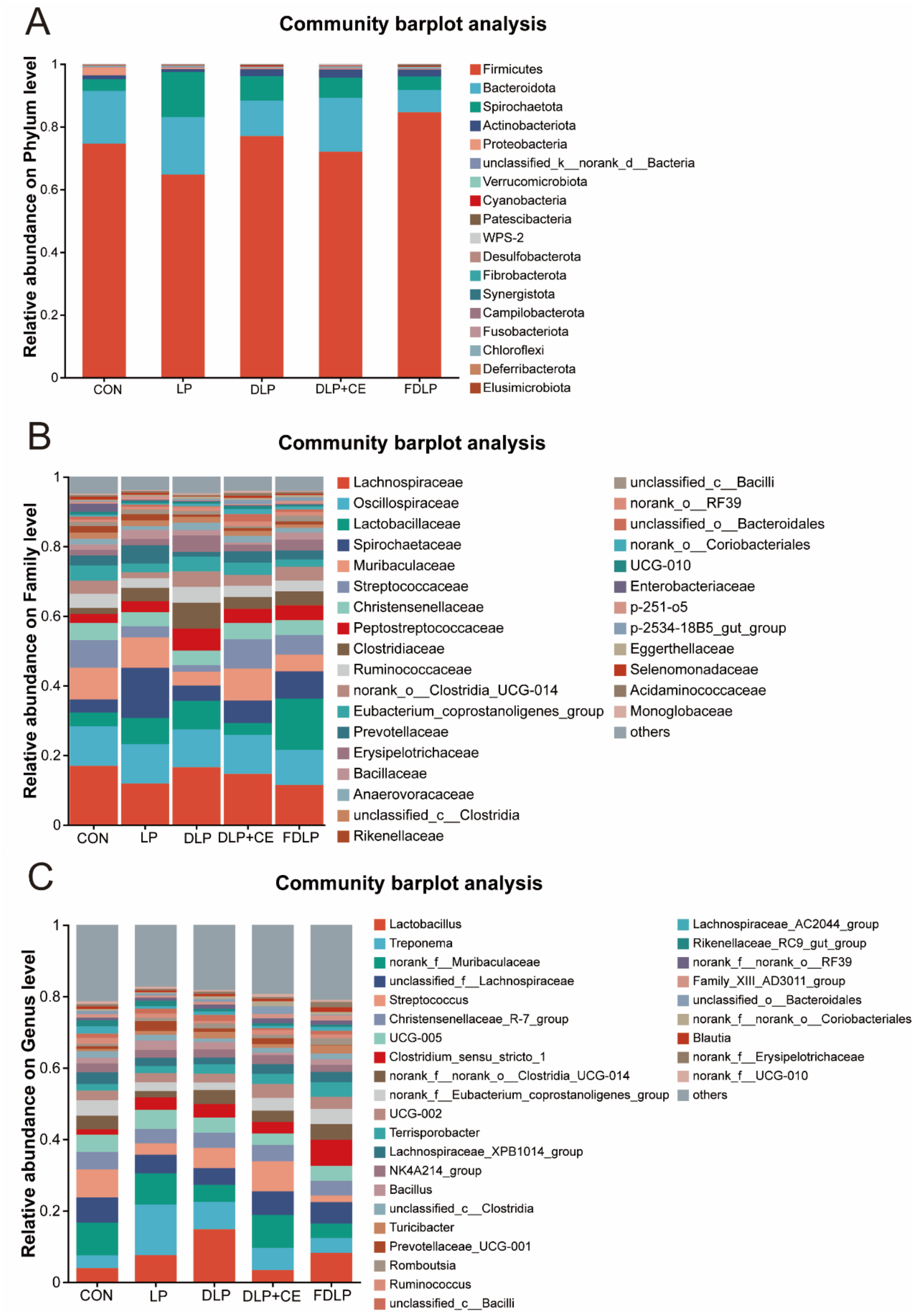

Figure S2 The relative abundances of bacteria at the phylum, family, and genus levels (n = 6). (**A**) Relative abundance of bacterial communities at the phylum level. (**B**) Relative abundance of bacterial communities at the family level. (**C**) Relative abundance of bacterial communities at the genus level. Abbreviations:

CON, control diet; LP, low-protein diet; DLP, diversified low-protein diet; DLP + CE, diversified low-protein diet with cellulase; FDLP, diversified low-protein diet with fermented feed.
